# Therapeutic Validity and Effectiveness of Preoperative Exercise on Functional Recovery after Joint Replacement: A Systematic Review and Meta-Analysis

**DOI:** 10.1371/journal.pone.0038031

**Published:** 2012-05-31

**Authors:** Thomas J. Hoogeboom, Ellen Oosting, Johanna E. Vriezekolk, Cindy Veenhof, Petra C. Siemonsma, Rob A. de Bie, Cornelia H. M. van den Ende, Nico L. U. van Meeteren

**Affiliations:** 1 Department of Rheumatology, Sint Maartenskliniek, Nijmegen, The Netherlands; 2 Department of Epidemiology, School for Public Health and Primary Care (Caphri), Maastricht University, Maastricht, The Netherlands; 3 Department of Physiotherapy, Gelderse Vallei Hospital, Ede, The Netherlands; 4 Department of Allied Health Care, NIVEL, Utrecht, The Netherlands; 5 Expertise Center Life Style, TNO, Leiden, The Netherlands; 6 Innovation Area Healthy for Life, TNO, Leiden, The Netherlands; 7 Center for Care Technology Research, Maastricht, The Netherlands; Ohio State University, United States of America

## Abstract

**Background:**

Our aim was to develop a rating scale to assess the therapeutic validity of therapeutic exercise programmes. By use of this rating scale we investigated the therapeutic validity of therapeutic exercise in patients awaiting primary total joint replacement (TJR). Finally, we studied the association between therapeutic validity of preoperative therapeutic exercise and its effectiveness in terms of postoperative functional recovery.

**Methods:**

(Quasi) randomised clinical trials on preoperative therapeutic exercise in adults awaiting TJR on postoperative recovery of functioning within three months after surgery were identified through database and reference screening. Two reviewers extracted data and assessed the risk of bias and therapeutic validity. Therapeutic validity of the interventions was assessed with a nine-itemed, expert-based rating scale (scores range from 0 to 9; score ≥6 reflecting therapeutic validity), developed in a four-round Delphi study. Effects were pooled using a random-effects model and meta-regression was used to study the influence of therapeutic validity.

**Results:**

Of the 7,492 articles retrieved, 12 studies (737 patients) were included. None of the included studies demonstrated therapeutic validity and two demonstrated low risk of bias. Therapeutic exercise was not associated with 1) observed functional recovery during the hospital stay (Standardised Mean Difference [SMD]: −1.19; 95%-confidence interval [CI], −2.46 to 0.08); 2) observed recovery within three months of surgery (SMD: −0.15; 95%-CI, −0.42 to 0.12); and 3) self-reported recovery within three months of surgery (SMD −0.07; 95%-CI, −0.35 to 0.21) compared with control participants. Meta-regression showed no statistically significant relationship between therapeutic validity and pooled-effects.

**Conclusion:**

Preoperative therapeutic exercise for TJR did not demonstrate beneficial effects on postoperative functional recovery. However, poor therapeutic validity of the therapeutic exercise programmes may have hampered potentially beneficial effects, since none of the studies met the predetermined quality criteria. Future review studies on therapeutic exercise should address therapeutic validity.

## Introduction

Total joint replacement is considered an effective and successful end-stage surgical procedure for relieving pain and improving functional status [1], [2]. However, a significant number of patients experience persistent pain and functional disability after major joint replacement [3], [4]. To enhance postoperative functional recovery, preoperative exercise is a potentially effective intervention by which to optimise the preoperative physical status of patients awaiting joint replacement [5], [6]. However, systematic reviews are inconclusive regarding the effectiveness of preoperative exercise in terms of postoperative health status following total hip (THR) or total knee replacement (TKR) [7]–[10].

These reviews might be flawed as they fail to take into account the therapeutic validity of the exercise interventions in the individual studies, as recommended by Herbert and Bø [11]. It is known that, in the field of preoperative therapeutic exercise, there is a tendency for trials to include relatively healthy patients [12], rather than patients with known high-risk profiles for delayed postoperative recovery (patients of older age [13], [14], with co-morbidities and/or poor pre-operative status [13]–[19]), thus excluding patients for whom preoperative exercise is specifically indicated [20]. Furthermore, to yield optimal effects, the content of an exercise programme should be in line with the latest research, be of sufficient volume [21], [22], and be tailored to the potential of the participants [23]. In terms of the latter, we hypothesize that poor therapeutic validity could result in negative study findings. To date, there is no clear set of criteria by which to assess the therapeutic validity of a therapeutic exercise intervention.

Therefore, the aim of our study was threefold. First, we developed a rating scale to assess the therapeutic validity of therapeutic exercise programmes. Second, we assessed the therapeutic validity of preoperative therapeutic exercise programmes in patients awaiting elective, primary THR or TKR, and, finally, we assessed the association between therapeutic validity and the effect of the interventions on postoperative functional recovery.

## Methods

The study comprised two phases: (1) a Delphi study to develop a rating scale for the therapeutic validity of therapeutic exercise, and (2) a systematic review and meta-analysis to assess the effectiveness of therapeutically valid exercise regimens in terms of observed functional recovery during the hospital stay, and in terms of self-reported and observed functioning after discharge within three months after surgery. This systematic review is reported in accordance with the Preferred Reporting Items for Systematic Reviews and Meta-Analyses (PRISMA) statement [24], [25].

### Delphi rounds

For the Delphi rounds, we followed the method described by Yates et al. (2005) [26]. For the Delphi panel, we selected five, internationally renowned, Dutch experts on therapeutic exercise. All participants met the following criteria: (1) previous involvement in a published RCT of a therapeutic exercise treatment, (2) two or more published articles on therapeutic exercise, (3) two or more conference presentations on therapeutic exercise, and (4) licensed health professional in a relevant discipline. The experts were invited by e-mail to participate in the study. Anonymity among experts was maintained throughout all Delphi rounds.

The Delphi study was conducted over four rounds [26]. In the first round, participants responded to open-ended questions regarding therapeutic validity of therapeutic exercise. We defined therapeutic validity as ‘*the potential effectiveness of a specific intervention given the potential target group of patients*’. In the second round, the first and second authors collated and grouped the responses from round one into a number of statements regarding different aspects of therapeutic validity in therapeutic exercise. The expert group was then asked to determine which of the statements would be essential in a rating scale designed to measure the therapeutic validity of therapeutic exercise programmes (one point = very unnecessary, through to seven points = very necessary). In the third round, the first author created personalised questionnaires for each of the experts, comprising the median and inter-quartile range (IQR) of scores of each statement (representing group level of agreement and the degree of consensus, respectively) and the rating of the individual expert as a reminder. All experts then reviewed and re-rated the statements. A list of statements, which achieved consensus agreement, was prepared by the first author. Consensus for inclusion was defined as a median rating of six or seven on the seven-point rating scale and an IQR of 1.5 or less [26]. In the fourth and final round, all experts were allowed to anonymously express any final concerns regarding the list. These concerns were either accepted or declined by the whole expert group. Finally, the first and second authors drafted the output generated by the Delphi panel into a workable rating scale for the therapeutic validity of exercise programmes.

### Systematic review

#### Search Strategy and Study Selection

We searched the following electronic databases (through to January 2012): MEDLINE (accessed by PubMed), Cochrane Central Register of Controlled Trials, EMBASE, ClinicalTrials.gov, CINAHL and PEDro. In addition, we manually searched the references of published studies. The initial search was not limited by language and comprised the terms *arthroplasty*, *exercise*, and related entry terms associated with a high-sensitivity strategy for the search of RCTs [27]. The complete search strategies used for the different databases are shown in Table S1.

We included (quasi)RCTs that compared the effectiveness of preoperative structured therapeutic exercise training with a control intervention, with postoperative recovery of functioning (self-reported or performance-based) as an outcome in patients older than 18 years awaiting elective, primary THR or TKR. Structured exercise training was defined as an intervention in which patients were engaged in planned and supervised exercise programmes (i.e. resistance, aerobic or functional exercise). We only included studies that reported means or differences between means, and respective dispersion values of postoperative functional recovery during the hospital stay and within 3 months after surgery. Exclusion criteria were: (1) duplicate publications or sub-studies of included trials, and (2) studies with two or fewer supervised exercise sessions. The comparator (control) group could be active (any non-exercise intervention) or placebo (no treatment or waiting list) group.

Titles and abstracts of retrieved articles were independently evaluated by two reviewers (TJH and JEV). Reviewers were not blinded to authors, institutions, or manuscript journals. Abstracts that did not provide enough information about the inclusion and exclusion criteria were retrieved for full-text evaluation. Reviewers independently evaluated full-text articles and determined eligibility for inclusion in review. Disagreements were resolved by consensus and, if disagreement persisted, by a third reviewer (C.H.M.E.). To avoid possible double counting of patients included in more than one report by the same authors or working groups, patient recruitment periods were evaluated and, if necessary, authors were contacted for clarification.

#### Data Extraction

Two reviewers (T.J.H. and E.O.) used standardised forms to independently extract the following information from each eligible publication: year of publication, geographical location, study population, functional outcome measures, duration of follow-up, and type and dose of exercise intervention. For the outcome measure of interest, the number of observations and means and standard deviations (SDs) were extracted for both the intervention and control groups at the following measurement points: 1) baseline (preoperative), 2) in-hospital (postoperative), and 3) after discharge (<3 months postoperative). If measures of variability were unavailable, we imputed the averaged SD of similar measures from other studies. If results were expressed as confidence intervals or interquartile ranges, we used transformation methods as recommended [28]. Where necessary, means and measures of dispersion were approximated from figures in the manuscripts using WebPlotDigitizer [29]. Characteristics of the exercise interventions were extracted, including the type, frequency, duration, and intensity. We used the Compendium of Physical Activities [21] to estimate the exercise intensity in terms of metabolic equivalents (METs). Exercise volume (total energy expenditure on exercise, in METs·h^−1^·wk^−1^) was calculated by multiplying the intensity in METs by total time spent exercising (number of exercise sessions multiplied by duration of each exercise session) [30].

Any disagreements about the extracted data were solved by consensus or by a third reviewer (C.H.M.E.). In case of missing data, the corresponding author of the included study was contacted.

#### Assessment of methodological (risk of bias) and therapeutic validity

Two reviewers (T.J.H and E.O.) independently assessed the methodological validity of the studies and the therapeutic validity of the therapeutic exercise programmes. The methodological validity (risk of bias) was scored using the adapted version of the Cochrane Collaboration's tool [31]. This adapted tool reviews five domains, with 11 items in total (see Table S2). Each item is rated as ‘yes’, ‘no’, or ‘unsure’. Studies fulfilling six or more items were regarded as having a low risk of bias [32]. Therapeutic validity was scored using the rating scale developed in the Delphi rounds. Each item was rated as ‘yes’ or ‘no’. Studies with six or more points out of nine were regarded as being of high therapeutic quality. Disagreements were resolved in a consensus meeting between the two raters. The strength of agreement between the two raters was measured by Cohen's κ coefficient (95%-confidence intervals), with κ = 0.41–0.60 indicating moderate agreement, κ = 0.61–0.80 representing good agreement, and κ≥0.81 representing very good agreement [33].

#### Data analysis

In this study, we compared structured, valid therapeutic exercise with a control intervention at three different outcome levels, namely 1) observed functional recovery during the hospital stay; 2) recovery of self-reported functioning within three months of surgery; and 3) recovery of observed functioning within three months of surgery. In our primary analyses, we only included highly valid studies (i.e. risk of bias score >6 & therapeutic validity score >5). Sensitivity analyses were performed without any restrictions on validity. All analyses were carried out separately for patients awaiting either TKR or THR. When more than one study was available, data were statistically pooled where appropriate.

Measures of functioning (performance and self-reported measures) in the treatment and control groups were transformed to standardised mean differences (Hedges *g*) to cope with the variety of outcome measures [28], [34]. To ensure uniform interpretability of all scales (i.e., higher scores representing more functional problems), we transformed our data according to the Cochrane recommendations [28]. For studies that compared multiple exercise interventions with a single control group, we split this shared control group into two or more subgroups with smaller sample sizes weighted in relation to different exercise interventions. We applied this approach to ensure reasonably independent comparisons and to overcome a unit-of-analysis error for studies that could contribute to multiple and correlated comparisons [28]. Calculations were performed using a random-effects model. An α value of <0.05 was considered statistically significant.

We assessed statistical heterogeneity of the treatment effect among studies using the inconsistency I^2^ test, in which values greater than 50% were considered indicative of high heterogeneity [28]. To assess heterogeneity between studies, we reran the meta-analyses whilst removing one study at a time to check if a particular study caused heterogeneity.

To explore whether effects of the exercise interventions on functional recovery were associated with therapeutic validity (0–9 points) or by exercise volume (METs·h^−1^·wk^−1^), we performed meta-regression analyses on each of the three outcome points (i.e. in-hospital functional recovery, short-term observed functional recovery, and short-term self-reported functional recovery), whilst accounting for hip or knee replacement. We evaluated the goodness of fit of each model using the adjusted *R^2^*, which denotes the proportion of between-study variation explained by the covariates.

Publication bias was assessed using a contour-enhanced funnel plot of each trial's effect size against the standard error [35]. Funnel plot asymmetry was evaluated by Begg and Egger tests, and a significant publication bias was considered to be present if the P value was less than 0.10. If publication bias was apparent, trim-and-fill computation was used to estimate the effect of publication bias on the interpretation of results [35], [36].

All analyses were conducted using Stata software, version 10.0 (Stata Inc., College Station, Texas).

## Results

### Delphi study

The initial open-ended questionnaire was sent to five experts in the field of therapeutic exercise, all of whom met our predetermined criteria. All five experts responded to the invitation and completed each of the four Delphi rounds; no attrition occurred. The experts agreed unanimously that trials on exercise therapy should be assessed on therapeutic validity and that therapeutic validity should be accounted for in best evidence synthesis in systematic reviews.

After the first round, a total of 49 unique statements were generated which could be aggregated into 10 recurrent themes (see Table S3). After the second round, consensus was reached on 22 out of the 49 statements (45%). The highest level of disagreement (i.e. largest IQR) was found for the item: “The exercise programme is personalised for each participant”. The lowest score was found for the item: “Natural fluctuations in disease activity must be controlled for.” In the third round, full consensus (i.e. median = 7 and IQR = 0) was not reached for any of the items, although for 10 items the degree of consensus was zero with a median score of six. In the fourth and final round, eight concerns were expressed regarding the pre-final list, mostly due to item formulation (n = 4).

In the final phase, the expert panel considered the 22 statements generated by the Delphi panel and collated them into a nine-item rating scale covering five critical areas. This scale was named the CONTENT (Consensus on Therapeutic Exercise Training) scale (see Table 1).

**Table 1:** The CONTENT scale for the therapeutic validity of therapeutic exercise programmes.

Items		Judgement	
**A. Patient eligibility**			
1.	Was the patient selection described?	Yes	No
	To score “yes”, patient selection should be described and participants should be screened for contraindications (for instance, using red and yellow flags) (this must be explicitly mentioned in the manuscript; otherwise “no”).		
2.	Was the patient selection adequate?	Yes	No
	This item can be scored as “yes” if:○ the goals of the therapeutic exercise match the participants' problems (for instance, if the goal of the therapeutic exercise is to improve a patients' functional status, then only patients with deprived functional status should be included). In this case participants' problems represent bodily functions and structures, activities and participation levels, see the 'International Classification of Functioning, Disability and Health (ICF); and○ the selection criteria match the majority of potential participants. Ergo, the therapeutic exercise should not be evaluated in a population that–in clinical practice–is nearly non-existent.		
**B. Competences and setting**			
3.	Were eligibility criteria for therapist and setting determined and adequate?	Yes	No
	The questions to be answered here are:○ Are the goals and content of the therapeutic exercise matched to the therapist's competences and skills?○ Are the goals and content of the therapeutic exercise matched to the location or setting where the therapeutic exercise takes place?If no eligibility criteria are described, this item should be scored as “no”.		
**C. Rationale**			
4.	Was the therapeutic exercise based on a-priori aims and intentions?	Yes	No
	Did the authors describe a-priori aims, intentions and hypotheses about the therapeutic exercise on theoretically driven and/or argued choices? If this question can be answered with “yes”, this item is scored as “yes”.		
5.	Was the rationale for the content and intensity of the therapeutic exercise described and plausible?	Yes	No
	Did the authors describe why they believed the content (e.g. resistance exercise training, aerobic exercise training, flexibility training, etc.) and intensity (e.g. moderate/vigorous intensity, length of exercise, etc.) of the studied intervention was likely to achieve their treatment goals?		
**D. Content**			
6.	Was the intensity of the therapeutic exercise described?	Yes	No
	This item can be scored as “yes” if:○ the content of the therapeutic exercise is described in specific terms (i.e. duration, frequency and intensity of exercise sessions (e.g. 80% VO_2max_, level of exertion (RPE), repetition maximum, etc.) and the total duration of the therapeutic exercise);○ the intensity of the therapeutic exercise was selected and adjusted on theoretically driven and/or argued choices; and○ the content of the therapeutic intervention is suitable for the majority of patients.		
7.	Was the therapeutic exercise monitored and adjusted when considered necessary?	Yes	No
	This item can be scored as “yes” if:1. the regular and structured monitoring of therapy progression allows the therapist to:○ strive for optimal exercise intensity;○ adjust the intervention in case of therapy failure on an individual level; and○ identify and monitor adverse events.2. the outcome measures match the therapy goals.		
8.	Was the therapeutic exercise personalised and contextualised to the individual participants?	Yes	No
	The goals and content of the therapeutic exercise should not only match the patients' bodily functions and structures, activities and participation levels, but also their personal and environmental factors (see ICF). This item can be scored as “yes” if the therapeutic exercise accounts for relevant personal (e.g. motivation, coping, ethnicity, etc.) and environmental (e.g. logistics, support family/friends, products and technology, etc.) factors for each of the included participants.		
**D. Adherence**			
9.	Was adherence to the therapeutic exercise determined and acceptable?	Yes	No
	For adherence to be properly described and acceptable, adherence should be described in such a way that it allows the reader to understand whether the actual executed therapeutic exercise differed from the planned therapeutic exercise (i.e. data should be provided on the achieved intensity, for example number of sessions attended, achieved exercise intensity, number of exercises etc.). Moreover, adherence should be quantitatively known, allowing it to be controlled for in the analysis.		

### Systematic review

#### Description of studies

**Figure 1:**
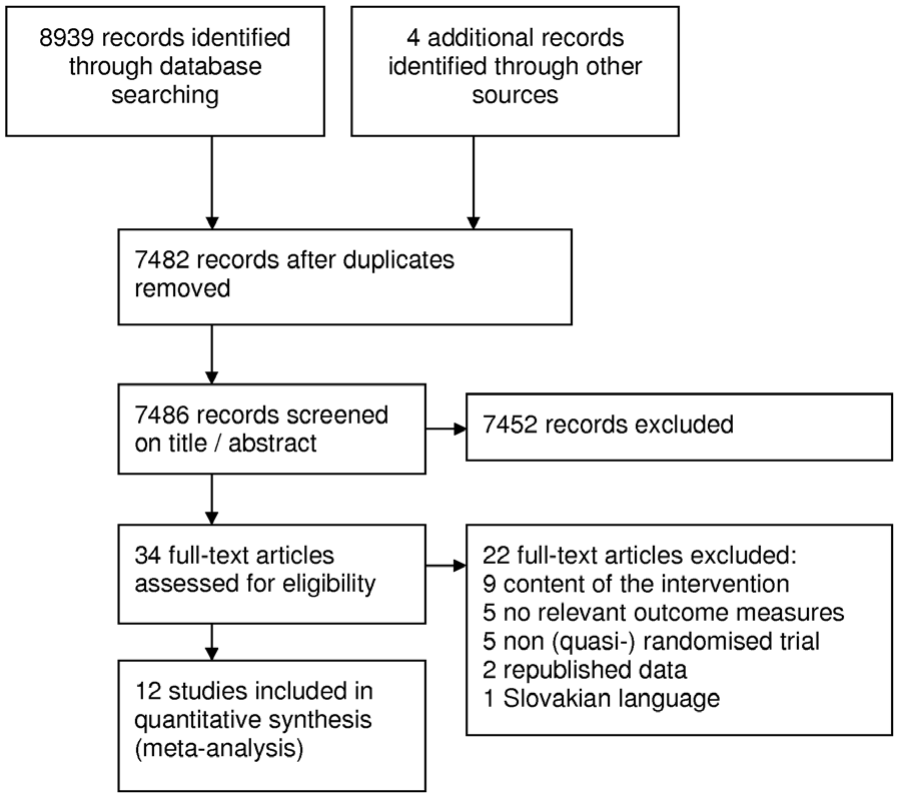
Selection of trials investigating preoperative exercise for total hip or knee replacement.

We identified a total of 8939 records in the initial search and removed 1457 duplicate publications. We excluded 7452 non-relevant records based on title or abstract screening. Full-text articles were retrieved for 34 publications and assessed for eligibility (Figure 1). Twelve English-language articles comprising 11 randomised controlled trials and one quasi-randomised controlled trial met the eligibility criteria [37]–[48]. One study presented data for both THR and TKR [45], therefore eight interventions on TKR and five interventions on THR were included. Moreover, one TKR study presented data for 2 comparisons [38], resulting in nine interventions in the TKR group. These 12 studies included a total of 737 patients (55% women), with a mean (SD) age of 66 (8) years and a Body Mass Index (BMI) of 31 (6).

The therapeutic exercise interventions prior to TKR and THR are described in Tables 2 and 3, respectively. Of the eight studies (n = 502) on therapeutic exercise prior to TKR, eight investigated resistance exercise [37]–[39], [44]–[48] and one investigated aerobic exercise [38]. Typically, these interventions were carried out 3 times a week for 5 weeks, at an intensity of 7.2 METs·h^−1^·wk^−1^ (see Table 2). Of the five studies (n = 235) on therapeutic exercise prior to THR, four studied resistance exercise [40]–[42], [45] and one examined functional exercise [43]. Typically, these interventions were carried out 2.5 times a week for a period of 6 weeks and at an intensity of 10.9 METs·h^−1^·wk^−1^ (see Table 3).

**Table 2:** Description of Supervised Exercise Intervention for patients awaiting Total Knee Replacement.

					Exercise group		Control group				Supervised exercise intervention				
Source, y	Study Location	Age, y†	Women,%	BMI†	No of sub- jects	Exercise intervention	No ofsub-jects	Controlintervention	Trial design	Deli- verer	Type	No. of times/wk	No. of weeks	Intensity	MET, h/wk‡
Beaupre et al, 2004	Canada	67 (6.5)	55	31.5 (5.5)	65	Resistance exercise	66	No intervention	RCT	PT	Cycling, lower extremity weight training	3	4	AT: 7.5 minutes at low intensity. WT: 3 sets, 10–15 repetitions , 5 exercises, intensity unclear (progressively increased to patients' tolerance).	6.6
D'Lima et al, 1996	USA	69 (5.5)	60	NA	10	Resistance exercise	10	Education session and leaflet	RCT	PT	Lower & Upper extremity weight training	3	6	WT: 45 minutes at intensity tolerated by patient (adjusted with one repetition every third day).	6.5
Evgeniadis et al, 2008	Greece	68.3 (3.5)	61	34.1 (5.0)	24	Resistance exercise	24	No intervention	RCT	PT+OS	Core & Upper extremity weight training	3	4	WT: 3–4 sets, 10–14 repetitions, ? exercises, intensity based on patient's ability to perform 1 set of 8 repetitions (progressed when 15 repetitions are performed comfortably).	5.3
Rodgers et al, 1998	USA	67.6 (18.4)	55	NA	12	Resistance exercise	11	No intervention	Quasi RCT	PT	Cycling, lower extremity weight training	3	6	AT: ? minutes at intensity according to baseline capacity. WT: ? sets, ? repetitions, 6 exercises, intensity unclear (adjusted after 3 weeks).	?
Rooks et al, 2006	USA	67.0 (8.2)	54	34.8 (7.9)	22	Resistance exercise	23	Education via leaflet and telephone	RCT	PT	Cycling, total body weight training (3 wks), aquatic training (3 wks)	3	6	AT: 10 minutes at moderate intensity. WT: 2 sets, 10 repetitions, 7 exercises, intensity tailored to person's fitness level and comfort with exercises (progression unclear).	10.0
Topp et al, 2009	USA	63.8 (6.8)	NA	32.1 (5.9)	26	Resistance exercise	28	No intervention	RCT	RES	Lower extremity weight training	3	4.3	WT: 2 sets, 10 repetitions, 9 exercises, intensity low (adjusted by increasing sets/resistance)	7.6
Weidenhielm et al, 1993	Sweden	63.5 (4.5)	52	29.6 (0.5)	20	Resistance exercise	20	No intervention	RCT	PT	Cycling, lower extremity weight training	3	5	AT: 10 minutes at 50 turns/min, unloaded. WT: 2 sets, 10 repetitions, 5 exercises, intensity against gravity to 3 kg (progression 10RM principle).	6.4
Williamson et al, 2007	UK	69.8 (9.4)	53	32.7 (6.1)	60	Resistance exercise	61	Education leaflet	RCT	PT	Lower extremity weight training	1	6	WT: 60 minutes, ? sets, ? repetitions, 9 exercises, intensity unclear (progression unclear).	4.3
D'Lima et al, 1996	USA	70.6 (6.5)	35	NA	10	Aerobic exercise	10	Education session and leaflet	RCT	PT	Cycling, arm ergometry, aquatic training	3	6	AT: 45 minutes at a heart rate of Resting Heart Rate+0.4–0.7·Heart Rate Reserve.	10.7
**Total**		66 (8)	54	32 (5)	249		253		8			2.8 (0.4)	5.3 (0.8)		7.2 (1.7)

Abbreviations: AT: Aerobic Training, BMI: Body Mass Index, MET: Metabolic Equivalent, OT: Occupational Therapist, PT: Physical Therapist, RCT: Randomised Clinical Trial, RES: Researcher, WT: Weight training.

† Values are expressed as mean (SD).

‡ Amount of energy expenditure per week during programmed exercise (1 metabolic equivalent equals 1 kcal·kg^−1^·hour^−1^).

**Table 3:** Description of supervised exercise intervention for patients awaiting total hip replacement.

					Exercise group		Control group				Supervised exercise intervention				
Source, y	Study Location	Age, y†	Women,%	BMI†	No of sub jects	Exercise intervention	No ofsub-jects	Control intervention	Trial design	Deli-verer	Type	No. oftimes/wk	No. Ofweeks	Intensity	MET, h/wk‡
Ferrara et al, 2008	Italy	63.4 (7.8)	61	NA	11	Resistance exercise	12	No intervention	RCT	PT	Cycling, lower extremity weight training	5	4	AT: 12.5 minutes at low/ moderate intensity. WT: 3– 4 sets, 8–12 repetitions, ? exercises, intensity unclear (progression unclear).	16.0
Gilbey et al, 2003	Australia	65.2 (11.1)	65	27.9 (4.3)	37	Resistance exercise	31	No intervention	RCT	PT	Cycling/arm ergometry/rowing, +aquatic training	2	8	AT: 5 min cycling/arm/ rowing (warming up), 10 min aquatic cycling/ running. WT: 1–3 sets, 10 repetitions, ? exercises. All intensity according to ACSM guidelines (sets increased when patient improved).	7.4
Rooks et al, 2006	USA	62.0 (9.7)	58	29.3 (7.4)	32	Resistance exercise	31	Education via telephone+leaflet	RCT	PT	Cycling, totalbody weight training (3 wks), aquatic training (3 wks)	3	6	AT: 10 minutes at moderate intensity. WT: 2 sets, 10 repetitions, 7 exercises, intensity tailored to person's fitness levelvand comfort with exercises (progression unclear).	10.0
Gocen et al, 2004	Turkey	51.3 (13.6)	36	26.3 (3.9)	30	Resistance exercise	30	No intervention	RCT	PT	Upper extremity weight training	0.5	8	Three times a day over an eight week period, patients performed 10 repetitions of ? exercises. A PT visited the patients at a two week interval.	?
Hoogeboom et al, 2010	the Netherlands	76.0 (4.2)	67	31.6 (11.3)	10	Functional exercise	11	One education session	Pilot RCT	PT	Walking, cycling, lower extremity weight training, functional training	2	4.5	AT: 5 min walk (warming up), 25 min cycling. WT: 1 set, 15 repetitions, 2 exercises. FT: 3 sets, 15 repetitions, 10 exercises. Intensity for all exercises was moderate to high (13– 14 RPE) and adjusted when patient's rated an exercise 12 RPE.	10.2
**Total**		62 (10)	56	28 (6)	120		115		5			2.5 (1.2)	6.1 (1.5)		10.9 (2.6)

Abbreviations: ACSM: American College of Sports Medicine, AT: Aerobic Training, BMI: Body Mass Index, MET: Metabolic Equivalent, OT: Occupational Therapist, PT: Physical Therapist, RCT: Randomised Clinical Trial, WT: Weight training.

† Values are expressed as mean (SD).

‡ Amount of energy expenditure per week during programmed exercise (1 metabolic equivalent equals 1 kcal·kg^−1^·hour^−1^).

#### Risk of Bias and Publication Bias assessment

Table S4 shows the methodological quality assessment of individual studies. The initial agreement of the reviewers on the total risk of bias assessment was 85% (112 of 132 items), and Cohen's Kappa (95%-CI) was 0.77 (0.67–0.85). All disagreements were resolved in a consensus meeting. Ten studies were assessed as having a high risk of bias and two studies were assessed as having a low risk of bias [37], [43]. The most prevalent limitations were found in items about blinding (patient, care provider, outcome assessor), allocation concealment, compliance and intention-to-treat analysis.

For the in-hospital recovery data, the Egger regression test suggested funnel plot asymmetry (*P* = 0.07), indicating publication bias. After applying the trim-and-fill procedure, we estimated that two studies were missing, and the adjusted estimate of overall SMD was −2.43 (95% CI, −3.77 to −1.08, *P*<0.01). Contour-enhanced funnel plots and statistical tests did not show any publication bias for the short-term post-operative observational data (Egger: *P* = 0.41 and Begg *P* = 0.54) and the self-reported data (Egger: *P* = 0.47 and Begg: *P* = 0.18).

#### Therapeutic validity assessment

Table S5 shows the therapeutic validity assessment score per individual study as assessed using the CONTENT scale. Cohen's kappa revealed a moderate agreement between the two raters of 0.70 (0.62–0.78); absolute agreement was 104 out of 117 items (89%). The item “Was the therapeutic exercise based on a-priori aims and intentions?” had the least agreement between the raters. All disagreements were resolved without consulting the third rater. The median score (IQR) and mean score (range) of the therapeutic quality of interventions was 1 (1) and 1.5 (0–5), respectively. None of the 13 interventions could be labelled as being therapeutically valid according to the cut-off score of six or higher. Both therapeutic validity and methodological validity scores are presented in Table 4.

**Table 4:** Methodological and therapeutic validity scores per study.

Study	Methodological Validity (0–11)	Therapeutic Validity (0–9)
Beaupre et al (2004)	7 (64%)	1 (11%)
D'Lima et al (1996) (RE)	3 (27%)	1 (11%)
D'Lima et al (1996) (AE)	3 (27%)	2 (22%)
Evgeniadis et al (2008)	4 (36%)	2 (22%)
Ferrara et al (2008)	5 (45%)	0 (0%)
Gilbey et al (2003)	2 (18%)	1 (11%)
Gocen et al (2004)	3 (27%)	0 (0%)
Hoogeboom et al (2010)	7 (64%)	5 (56%)
Rodgers et al (1998)	2 (18%)	2 (22%)
Rooks et al (2006)	4 (36%)	3 (33%)
Topp et al (2009)	3 (27%)	2 (22%)
Weidenhielm et al (1993)	4 (36%)	0 (0%)
Williamson et al (2007)	4 (36%)	1 (11%)

Abbreviations: AE = Aerobic exercise, RE = Resistance exercise.

The categories ‘Setting and Therapist’, ‘Monitoring’, and ‘Adherence’ had the lowest score; none of the interventions included these aspects in their intervention. The highest-scoring category was ‘Rationale of the study’, with nine out of 13 studies scoring ‘Yes’ (69%). Two studies (15%) provided a rationale for the content of the therapy. Patient selection was described in four interventions (31%), but only one intervention (8%) was in line with the described aims and intentions of the intervention. Intensity of the intervention was described adequately in three of the 13 interventions (23%).

#### Association between intervention and in-hospital functional recovery

**Figure 2:**
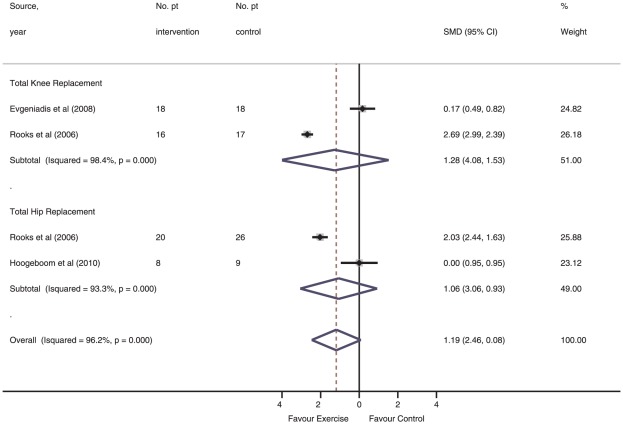
Functional recovery during hospital stay in individual studies of structured exercise training vs. control intervention.

None of the three studies (132 patients) in this category met the requirements for methodological and therapeutic validity [39], [43], [45]. Sensitivity analysis of the overall pooled effect of structured preoperative exercise vs. control in terms of functional recovery during the hospital stay was −1.19 (95% CI, −2.46 to 0.08; *I* ^2^, 96.2%; *P* for heterogeneity <0.001) (Figure 2). Similar pool effects were found when the analysis was separated into THR [43], [45] and TKR [40], [45], albeit with broader 95% confidence intervals (Figure 2). Meta-regression did not demonstrate an association between the pooled effect and exercise volume (β = −1.70; 95%-CI −21.56–18.15)) or therapeutic validity score (β = 0.32; 95%-CI −13.23–13.87)).

#### Association between intervention and short-term observed functional recovery

**Figure 3:**
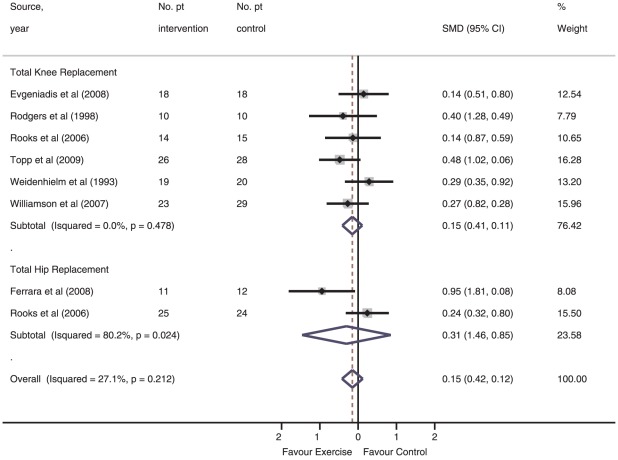
Short-term recovery of observed functioning in individual studies of structured exercise vs. control intervention.

None of the seven studies in this category met the requirements for methodological or therapeutic validity [39], [40], [44]–[48]. Disregarding any predetermined validity scores, sensitivity analyses found that overall short-term observed functional status was not associated with structured exercise; SMD −0.15 (95% CI, −0.42 to 0.12; *I* ^2^, 27.1%, *P* for heterogeneity = 0.212) (Figure 3). For the TKR subgroup (6 studies, 230 patients) [39], [44]–[48], random-effect modelling revealed a non-significant SMD for the effect of structured exercise on observed functional recovery, SMD −0.15 (95% CI, −0.41 to 0.11; *I* ^2^, 0.0%, *P* for heterogeneity = 0.478). For the THR subgroup (2 studies, 72 patients) [40], [45], a non-significant SMD of −0.31 (95% CI, 1.46 to 0.85, *I* ^2^, 80.2%, *P* for heterogeneity = 0.024) was found for the effect of structured preoperative exercise on observed functional recovery. Meta-regression demonstrated no association between the interventions' short-term effects on functional recovery and exercise volume (β = −0.15; 95%-CI −.364–0.07) or therapeutic validity (β = 0.08; 95%-CI −0.09–0.26).

#### Association between intervention and short-term self-reported functional recovery

**Figure 4:**
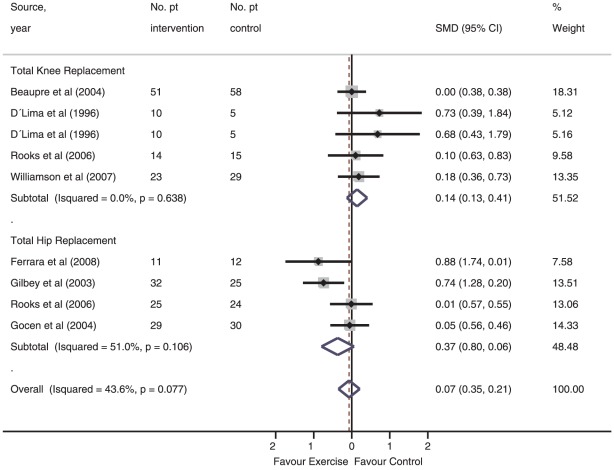
Short-term recovery of self-reported functioning in individual studies of structured exercise vs. control intervention.

Methodological validity was demonstrated in one of the seven studies in this category [37], while therapeutic validity was found in none. Sensitivity analysis of the seven studies comparing structured exercise (205 patients) vs. control (203 patients) [37], [38], [40]–[42], [45], [48], showed that exercise was not associated with self-reported short-term functional recovery after major joint replacement; SMD −0.07 (95% CI, −0.35 to 0.21; *I* ^2^, 43.6%, *P* for heterogeneity = 0.077) (Figure 4). For the TKR subgroup [37], [38], [45], [48], the overall association between five structured therapeutic exercise programmes vs. control and short-term self-reported functioning was 0.14 (95% CI, −0.13 to 0.41; *I* ^2^, 0.0%, *P* for heterogeneity = 0.638). For the THR subgroup [40]–[42], [45], random-effect models of four studies (188 patients) on structured exercise revealed a non-significant SMD in favour of structured exercise; SMD −0.37 (95% CI, −0.80 to 0.06; I2, 51.0%, *P* for heterogeneity = 0.106). Meta-regression showed no association between pooled effects and exercise volume (β = 0.02; 95%-CI −0.15–0.19)) or therapeutic validity (β = −0.01; 95%-CI −0.18–0.15)).

## Discussion

Our results demonstrate that the effectiveness of (highly) valid, structured therapeutic exercise training in individuals awaiting major joint replacement surgery remains unconfirmed. Of the 12 eligible studies, only two met the requirements for methodological quality and none met the prespecified requirements for therapeutic validity, highlighting a lack of quality in this field. Furthermore, pooling data from all eligible studies showed no benefit of preoperative therapeutic exercise therapy in terms of functional recovery after THR or TKR. These findings should, however, be interpreted with caution.

Expert opinion in our Delphi rounds identified five critical areas, comprising a total of 9 items, as being important for the therapeutic validity of a therapeutic exercise intervention. These five critical areas are patient selection, therapist and setting selection, rationale, content, and adherence, and are supported by evidence from the literature. For example, several studies have demonstrated that adequate patient selection can be of great importance in treatment effectiveness, as some patients respond differently to non-pharmacological interventions than others [49]–[51]. Thus, proper patient selection might result in greater therapy gains [52]. In addition, the selection of therapist and setting are also both known to influence treatment effects [53]. Furthermore, a plausible rationale regarding the benefits of the therapeutic exercise programme–especially if there is little or no previous experience with the intervention–is thought to be necessary to achieve therapy effects [54]. In fact, studies lacking a clear rationale are even considered to be unethical [55]. Adequate intervention content, characterised by sufficient dosing based on theoretical or argued choices, monitoring and personalisation, is perhaps the most important factor in yielding therapy effects. For example, evidence shows that strength training programmes produce the greatest increases in muscle strength if the training load is high [22] without the consideration of frailty [56]. The use of intermediate outcomes is also essential to optimally dose the therapeutic exercise intervention, to achieve therapy progress, and to prevent therapy failure [57]. Finally, the last critical area identified by the Delphi group was adherence to the intervention. Adherence to the exercise programme determines the extent to which therapy dosing is indeed achieved [58]. Therefore, it has been recommended that exercise programmes should be described in sufficient detail to enable readers to understand how the intervention was actually carried out [11]. In conclusion, each of the five aspects of therapeutic validity identified by the Delphi study is supported by the literature.

Our finding that preoperative therapeutic exercise has no beneficial effect on functional recovery after joint replacement surgery is in line with our hypothesis that suboptimal therapeutic exercise elicits no effect. None of the included studies met the predetermined requirements for therapeutic validity. An apposite example demonstrating this lack of therapeutic validity is that, although nine out of 13 exercise interventions provided a rationale for why preoperative exercise would elicit beneficial effects, only one group [43] actually applied their rationale to their patient selection criteria (i.e. by including patients with a high risk of delayed functional recovery), and only two studies [43], [45] applied this rationale to their exercise programme (i.e. by selecting their exercise dosing accordingly). Moreover, none of the included interventions monitored therapy dosing to achieve and maintain optimal exercise dosing [57], as is further illustrated by the finding that only three studies [38], [40], [43] reported a supervised exercise dose greater than the regularly prescribed weekly amount of physical activity (i.e. 10 METs·h^−1^·wk^−1^) [59]. Finally, adherence was often not, or only marginally, reported. Apart from the number of attended sessions, authors should provide information on the prespecified exercise protocol and whether the intended exercise intensity was reached. In conclusion, we recommend that future studies on preoperative therapeutic exercise develop a highly valid therapy protocol, for which our rating scale could be used as a blueprint.

For an exercise programme to be considered therapeutically valid, we arbitrarily chose a cut-off value of six out of nine items on the CONTENT scale. Lowering the cut-off score to five or even four points would not have altered the our conclusions regarding short-term postoperative functional recovery. Regarding the in-hospital functional recovery, lowering the cut-off score to four or five would have identified one pilot trial [43] that was insufficiently powered to assess differences in postoperative recovery. Whether the current cut-off value represents a true threshold for therapeutic validity needs to be further investigated.

Ten out of 12 studies were considered to have a high risk of bias. Allocation concealment and blinding were the lowest scoring items in the risk of bias assessment. Because most of the studies lack allocation concealment, readers should be aware that these studies are more susceptible to selection bias, and this may affect the generalisability of our results. Moreover, given that most studies were insufficiently blinded and that the majority of studies did not use intention-to-treat analysis, the apparent results of our meta-analysis may have been inflated [60], [61].

Since effectiveness in randomised trials depends on the quality of the intervention, the lack of criteria to assess this quality is surprising. To date, some systematic reviews have investigated the relationship between exercise intensity and therapeutic effectiveness post-hoc [30], [62], with varying effects. One limitation of our study is that we were unable to draw conclusions regarding the validity of our rating scale, as none of the included studies could be classified as being highly valid. In fact, the majority of the interventions scored in the lowest tertile of the scale, preventing us from evaluating the relationship between therapy outcomes and therapy validity. Another limitation is that the CONTENT-scale might not only evaluate the therapeutic validity of an exercise program but also how well the exercise program was justified and how completely the justification was reported. Perhaps some of the studies employed adequate exercise programs but scored poorly on the scale because the study reports did not include a complete justification of the exercise programs.

So far, several systematic reviews [7], [8], [63], narrative reviews [9], [64], and meta-analyses [10], [65] have been published on preoperative exercise in patients awaiting joint replacement, but none of these reviews assessed the quality of the included interventions [11]. Taken the therapeutic validity into account, we have reached a similar conclusion to previous reviews, namely that the current intervention studies, which is mainly of low methodological validity, does not show that therapeutic exercise has beneficial effects on postoperative outcomes. However, what our review adds is that readers should also take the low therapeutic validity into consideration when interpreting these conclusions. Future studies should therefore specifically aim to include patients at need, that is those at risk for postoperative delayed recovery (based on a validated clinical decision rule) [52], provide a (piloted) [23] therapeutically sound and feasible exercise programme of sufficient, titrated dosing [57] and evaluated on relevant and amendable parameters (for instance heart rate recovery) [66]. The preoperative exercise program for patients awaiting coronary artery bypass grafting reported by Hulzebos et al (2006) is an illustration of the systematic development of an exercise program while addressing critical areas for therapeutic validity [20].

In conclusion, none of the 13 included therapeutic exercise programmes met our predetermined criteria for high therapeutic validity, making it unlikely that the interventions evaluated in these studies would have elicited relevant effects. In our view, the interpretation and development of therapeutic exercise programmes would be facilitated if international consensus could be reached on a select number of mandatory criteria for therapeutic validity. Finally, we recommended that future review studies on therapeutic exercise should not only determine the methodological validity, but also the therapeutic validity of the included trials.

## Supporting Information

Table S1 Full bibliography of the electronic searches.
(DOCX)

Table S2 Assessment of risk of bias scale.
(DOCX)

Table S3 Summary of the statements generated by the Delphi panel.
(DOCX)

Table S4 Assessment of risk of bias per individual study per scale item.
(DOCX)

Table S5 Assessment of therapeutic validity per individual study per scale item.
(DOCX)
