# Supplementary material for: Therapeutic Validity and Effectiveness of Preoperative Exercise on Functional Recovery after Joint Replacement: A Systematic Review and Meta-Analysis
Source: PLoS One. 2012 May 31;7(5):e38031. doi: 10.1371/journal.pone.0038031 (PMC3364996; doi:10.1371/journal.pone.0038031)
Supplement: Table S1 — Full bibliography of the electronic searches. (DOCX) [file pone.0038031.s001.docx]

**Table S1.** Full bibliography of the electronic searches.

#### Search History PUBMED

| **ID** | **SEARCH** | **HITS** |
| --- | --- | --- |
| #12 | #5 AND #8 AND #11 | **1469** |
| #11 | #9 NOT #10 | 2489722 |
| #10 | (animals[MeSH Terms]) NOT humans[MeSH Terms] | 3629850 |
| #9 | randomized controlled trial[Publication Type]) OR controlled clinical trial[Publication Type]) OR randomized[Title/Abstract]) OR placebo[Title/Abstract]) OR drug therapy[MeSH Subheading]) OR randomly[Title/Abstract]) OR trial[Title/Abstract]) OR groups[Title/Abstract] | 2908964 |
| #8 | exercise[MeSH Terms]) OR exercise therapy[MeSH Terms]) OR hydrotherapy[MeSH Terms]) OR physical fitness[MeSH Terms]) OR physical therapy[MeSH Terms]) OR physical therapy modalities[MeSH Terms]) OR rehabilitation[MeSH Terms]) OR gymnastics[MeSH Terms]) OR exercise*[Text Word]) OR training*[Text Word]) OR gymnast*[Text Word]) OR exercise therapy[Text Word]) OR hydrotherap*[Text Word]) OR water therap*[Text Word]) OR swim*[Text Word]) OR rehabilitation*[Text Word]) OR physical therap*[Text Word]) OR physiotherapy[Text Word] | 769712 |
| #5 | arthroplasty OR joint prosthesis OR arthroplasty, replacement, hip OR arthroplasty, replacement, knee [MeSH Terms]) OR hip prosthesis[MeSH Terms]) OR knee prosthesis[MeSH Terms]) OR arthroplasty[Text Word]) OR total joint*[Text Word]) OR total hip*[Text Word]) OR total knee*[Text Word] | 61631 |

#### Search string Embase

| **ID** | **SEARCH** | **HITS** |
| --- | --- | --- |
| #18 | #7 AND #13 AND #17 | **3053** |
| #17 | 'exercise'/exp OR 'exercise' OR 'kinesiotherapy'/exp OR 'kinesiotherapy' OR 'hydrotherapy'/exp OR 'hydrotherapy' OR 'physiotherapy'/exp OR 'physiotherapy' OR 'fitness'/exp OR 'fitness' OR 'physical education'/exp OR 'physical education' OR 'training'/exp OR 'training' OR 'physical therapy'/de OR 'physical therapy' OR 'gymnastics'/de OR 'gymnastics' OR 'exercise therapy'/de OR 'exercise therapy' | 698107 |
| #13 | #11 NOT #12 | 13217635 |
| #12 | 'animal'/exp NOT 'human'/exp | 1666924 |
| #11 | 'randomized controlled trial'/exp OR 'randomized controlled trial' OR 'controlled clinical trial'/exp OR 'controlled clinical trial' OR 'randomized' OR 'placebo'/de OR 'placebo' OR 'drug therapy'/exp OR 'drug therapy' OR 'randomly' OR 'trial' OR 'groups' | 14625836 |
| #7 | 'arthroplasty'/exp OR 'arthroplasty' OR 'arthroplasty'/de OR 'joint prosthesis'/exp OR 'joint prosthesis' OR 'joint prosthesis'/de OR 'hip arthroplasty'/exp OR 'hip arthroplasty' OR 'hip arthroplasty'/de OR 'knee arthroplasty'/exp OR 'knee arthroplasty' OR 'knee arthroplasty'/de OR 'total knee replacement'/exp OR 'total knee replacement' OR 'total knee replacement'/de OR 'knee prosthesis'/exp OR 'knee prosthesis' OR 'knee prosthesis'/de OR 'hip prosthesis'/exp OR 'hip prosthesis' OR 'hip prosthesis'/de OR 'total hip prosthesis'/exp OR 'total hip prosthesis' OR 'total hip prosthesis'/de OR 'total joint replacement'/de OR 'total joint replacement' OR 'total joint prosthesis'/de OR 'total joint prosthesis' | 73551 |

#### Search string Central (Cochrane Clinical Trials)

| **ID** | **SEARCH** | **HITS** |
| --- | --- | --- |
| #1 | "accession number" near pubmed in Clinical Trials | 377640 |
| #2 | "accession number" near embase in Clinical Trials | 118524 |
| #3 | (**#1** OR **#2**) | 434845 |
| #4 | arthroplasty:kw OR arthroplasty OR joint prosthesis:kw OR knee prosthesis:kw OR hip prosthesis:kw OR arthroplasty near replacement* OR total near joint* OR total near hip* OR total near knee* OR hip near replacement OR knee near replacement in Clinical Trials | 5092 |
| #5 | exercise:kw OR exercise therapy:kw OR hydrotherapy:kw OR physical therapy:kw OR physical fitness:kw OR physical therapy modalities:kw OR rehabilitation:kw OR gymnastics:kw OR exercise* OR hydrotherap* OR water near therap* OR swim* OR rehabilitation* OR physical near therap* OR physiotherapy in Clinical Trials | 53282 |
| #6 | (#4 AND #5) | 701 |
| #7 | (#6 AND NOT #3) | **38** |

#### Search string CINAHL

| **ID** | **Search** | **Hits** |
| --- | --- | --- |
| S6 | #S1 AND #S2 AND #S5 | **611** |
| S5 | ( #S3 NOT #S4 ) | 237365 |
| S4 | (MH "Animals") NOT (MM "Human") | 21209 |
| S3 | (PT Randomized controlled trial) OR (PT Clinical trials) OR (TX randomized) OR (TX placebo) OR (MM "Drug therapy") OR (TX randomly) OR (TX trial) OR (TX groups) | 239922 |
| S2 | (MM "Exercise+") OR (MM "Therapeutic exercise+") OR (MM "Physical fitness+") OR (MM "Physical therapy+") OR (MM "Hydrotherapy+") OR (MM "Rehabilitation+") OR (MM "Gymnastics+") OR (TX exercise*) OR (TX training*) OR (TX exercise N5 therapy) OR (TX water N5 therapy) OR (TX rehabilitation) OR (TX hydrotherap*) OR (TX physiotherapy) | 284449 |
| S1 | (MM "Arthroplasty+") OR (MM "Arthroplasty, Replacement+") OR (MM "Arthroplasty, Replacement, Hip+") OR (MM "Arthroplasty, Replacement, Knee+") OR (TX Arthroplasty) OR (TX hip N5 prosthesis) OR (TX knee N5 prosthesis) OR (TX total joint*) OR (TX total knee*) OR (TX total hip*) | 11585 |

#### Search string Web of Science

| **ID** | **SEARCH** | **HITS** |
| --- | --- | --- |
| # 7 | #6 AND Document Type=(Article)  Databases=SCI-EXPANDED, SSCI, A&HCI, CPCI-S, CPCI-SSH Timespan=All Years | **3091** |
| # 6 | #1 AND #2 AND #5  Databases=SCI-EXPANDED, SSCI, A&HCI, CPCI-S, CPCI-SSH Timespan=All Years | 3367 |
| # 5 | #3 NOT #4  Databases=SCI-EXPANDED, SSCI, A&HCI, CPCI-S, CPCI-SSH Timespan=All Years | 3247611 |
| # 4 | TS=(animals) NOT TS=(humans)  Databases=SCI-EXPANDED, SSCI, A&HCI, CPCI-S, CPCI-SSH Timespan=All Years | 525626 |
| # 3 | TS=(randomized controlled trial OR controlled clinical trial OR randomized OR placebo OR drug therapy OR randomly OR trial OR groups)  Databases=SCI-EXPANDED, SSCI, A&HCI, CPCI-S, CPCI-SSH Timespan=All Years | 3382798 |
| # 2 | TS=(exercise OR exercise therap* OR hydrotherap* OR physical fitness OR physical therap* OR rehabilitation OR gymnastic* OR training* OR physiotherap* OR water therap* OR swim*)  Databases=SCI-EXPANDED, SSCI, A&HCI, CPCI-S, CPCI-SSH Timespan=All Years | 674360 |
| # 1 | TS=(arthroplasty OR joint prosthesis OR joint replacement OR knee replacement OR hip replacement OR hip prosthesis OR knee prosthesis OR total joint* OR total hip* OR total knee*)  Databases=SCI-EXPANDED, SSCI, A&HCI, CPCI-S, CPCI-SSH Timespan=All Years | 88640 |

#### Search string PEDro

| **id** | **search** | **hits** |
| --- | --- | --- |
| #1 | *arthroplasty AND “clinical trial” | **172** |
| #2 | *prosthesis AND “clinical trial” | **37** |
| #3 | *replacement AND “clinical trial” | **209** |

**Search string Clinical trials (**[**www.clinicaltrials.gov**](http://www.clinicaltrials.gov/)**)**

| **ID** | **Search** | **hits** |
| --- | --- | --- |
| #1 | arthroplasty OR total hip replacement OR total knee replacement OR replacement OR prosthesis | 2591 |
| #2 | exercise OR “exercise therapy” OR hydrotherapy OR “physical fitness” OR “physical therapy” OR training OR physiotherapy OR rehabilitation | 21643 |
| #3 | #1 AND #2 | **259** |
