# Supplementary material for: Therapeutic Validity and Effectiveness of Preoperative Exercise on Functional Recovery after Joint Replacement: A Systematic Review and Meta-Analysis
Source: PLoS One. 2012 May 31;7(5):e38031. doi: 10.1371/journal.pone.0038031 (PMC3364996; doi:10.1371/journal.pone.0038031)
Supplement: Table S3 — Summary of the statements generated by the Delphi panel. (DOCX) [file pone.0038031.s003.docx]

**Table S3.** Summary of the statements generated by the Delphi panel.

|  | **Round 1** | **Round 2** | **Round 3 & 4** |
| --- | --- | --- | --- |
|  | number of items | Consensus | Consensus |
| Patient characteristics | 6 | 4 | 3 |
| Therapist eligibility | 4 | 3 | 2 |
| Therapy setting | 1 | 1 | 1 |
| Exercise intensity | 8 | 3 | 4 |
| Therapy content | 8 | 4 | 4 |
| Co-interventions | 4 | 0 | 0 |
| Therapy monitoring | 8 | 4 | 4 |
| Therapy evaluation | 4 | 1 | 1 |
| Follow-up time | 2 | 0 | 0 |
| Exercise adherence | 4 | 3 | 3 |
| Total | 49 | 23 | 22 |
