# Supplementary material for: Therapeutic Validity and Effectiveness of Preoperative Exercise on Functional Recovery after Joint Replacement: A Systematic Review and Meta-Analysis
Source: PLoS One. 2012 May 31;7(5):e38031. doi: 10.1371/journal.pone.0038031 (PMC3364996; doi:10.1371/journal.pone.0038031)
Supplement: Table S4 — Assessment of risk of bias per individual study per scale item. (DOCX) [file pone.0038031.s004.docx]

**TableS4.** Assessment of risk of bias per individual study per scale item.

|  |  |  |  | Blinding |  |  |  |  |  |  |  |  |
| --- | --- | --- | --- | --- | --- | --- | --- | --- | --- | --- | --- | --- |
| Study | Adequate randomi-sation | Allocation concealed | Patient | Care provider | Outcome assessor | Drop-out rate described | Intention to treat analysis | Groups similar at baseline | Cointer-ventions avoided | Com-pliance acceptable | Timing of outcome assessment similar | Total score |
| Beaupre *et al* (2004) | Yes | Unsure | No | No | Unsure | Yes | Yes | Yes | Yes | Yes | Yes | 7 |
| D’Lima *et al* (1996) | Yes | Unsure | No | No | No | No | Unsure | Unsure | Yes | Unsure | Yes | 3 |
| Evgeniadis *et al* (2008) | Yes | Unsure | No | No | No | No | Yes | Yes | Unsure | Unsure | Yes | 4 |
| Ferrara *et al* (2008) | Yes | Unsure | No | No | No | Yes | Unsure | Yes | Yes | Unsure | Yes | 5 |
| Gilbey *et al* (2003) | Unsure | Unsure | No | No | No | No | Unsure | Unsure | Unsure | Yes | Yes | 2 |
| Gocen *et al* (2004) | Yes | Unsure | No | No | No | Yes | Unsure | No | Yes | Unsure | No | 3 |
| Hoogeboom *et al* (2010) | Yes | Yes | No | No | Unsure | No | Yes | Yes | Yes | Yes | Yes | 7 |
| Rodgers *et al* (1998) | No | No | No | No | No | Yes | Unsure | Unsure | Yes | Unsure | No | 2 |
| Rooks *et al* (2006) | Unsure | Unsure | No | No | No | No | Yes | Yes | Unsure | Yes | Yes | 4 |
| Topp *et al* (2009) | Unsure | Unsure | No | No | No | Yes | Unsure | Unsure | Yes | No | Yes | 3 |
| Weidenhielm *et al* (1993) | Yes | Unsure | No | No | No | Yes | Unsure | Unsure | Yes | Unsure | Yes | 4 |
| Williamson *et al* (2007) | Yes | Yes | No | No | Unsure | No | Unsure | Yes | No | Unsure | Yes | 4 |
| Total score | 8 (67%) | 2 (17%) | 0 (0%) | 0 (0%) | 0 (0%) | 6 (50%) | 4 (33%) | 6 (50%) | 8 (67%) | 4 (%33) | 10 (83%) |  |
