# Supplementary material for: Therapeutic Validity and Effectiveness of Preoperative Exercise on Functional Recovery after Joint Replacement: A Systematic Review and Meta-Analysis
Source: PLoS One. 2012 May 31;7(5):e38031. doi: 10.1371/journal.pone.0038031 (PMC3364996; doi:10.1371/journal.pone.0038031)
Supplement: Table S5 — Assessment of therapeutic validity per individual study per scale item. (DOCX) [file pone.0038031.s005.docx]

| **Table S5**. Assessment of therapeutic validity per individual study per scale item. | | | | | | | | | | | |
| --- | --- | --- | --- | --- | --- | --- | --- | --- | --- | --- | --- |
|  | Patient eligibility | |  | Rationale | |  | Content | | |  |  |
| **Study** | Described | Adequate | Setting and therapist | Study | Intervention |  | Intensity | Monitored | Personalized | Adherence | Total score |
| Beaupre *et al* (2004) | no | no | no | yes | no |  | no | no | no | no | 1 (11%) |
| D’Lima *et al* (1996) | no | no | no | yes | no |  | no | no | no | no | 1 (11%) |
| D’Lima *et al* (1996) | no | no | no | yes | no |  | yes | no | no | no | 2 (22%) |
| Evgeniadis *et al* (2008) | yes | no | no | yes | no |  | no | no | no | no | 2 (22%) |
| Ferrara *et al* (2008) | no | no | no | no | no |  | no | no | no | no | 0 (0%) |
| Gilbey *et al* (2003) | no | no | no | no | no |  | yes | no | no | no | 1 (11%) |
| Gocen *et al* (2004) | no | no | no | no | no |  | no | no | no | no | 0 (0%) |
| Hoogeboom *et al* (2010) | no | yes | no | yes | yes |  | yes | no | yes | no | 5 (56%) |
| Rodgers *et al* (1998) | yes | no | no | yes | no |  | no | no | no | no | 2 (22%) |
| Rooks *et al* (2006) | yes | no | no | yes | yes |  | no | no | no | no | 3 (33%) |
| Topp *et al* (2009) | yes | no | no | yes | no |  | no | no | no | no | 2 (22%) |
| Weidenhielm *et al* (1993) | no | no | no | no | no |  | no | no | no | no | 0 (0%) |
| Williamson *et al* (2007) | no | no | no | yes | no |  | no | no | no | no | 1 (11%) |
| Total score | 4 (31%) | 1 (8%) | 0 (0%) | 9 (69%) | 2 (15%) |  | 3 (23%) | 0 (0%) | 1 (8%) | 0 (0%) |  |
